# Primary postpartum hemorrhage and associated factors among delivering women in Gedeo Zone, Southern Ethiopia

**DOI:** 10.3389/fmed.2023.1096501

**Published:** 2023-02-14

**Authors:** Getachew Assefa Zenebe, Wagaye Alemu Zenebe, Temesgen Muche Ewunie, Selamawit Dires

**Affiliations:** ^1^Department of Public Health, School of Public Health, College of Medicine and Health Sciences, Dilla University, Dilla, Ethiopia; ^2^Department of Human Nutrition, School of Public Health, College of Medicine and Health Sciences, Dilla University, Dilla, Ethiopia; ^3^Department of Reproductive Health, School of Public Health, College of Medicine and Health Sciences, Dilla University, Dilla, Ethiopia

**Keywords:** primary postpartum hemorrhage, vaginal bleeding, delivering women, Gedeo Zone, Southern Ethiopia

## Abstract

**Introduction:**

Primary postpartum hemorrhage is still the main cause of maternal death worldwide, especially in low-resource nations like Ethiopia where there are insufficient healthcare facilities and a shortage of skilled medical personnel. Data on the prevalence of primary postpartum hemorrhage in the study population are scarce or non-existent.

**Objective:**

The aim of this study was to assess the prevalence of primary postpartum hemorrhage and its associated factors among delivering women in Gedeo Zone, Southern Ethiopia, in 2021.

**Methods:**

A facility-based cross-sectional study was carried out from January 1 to March 30, 2021, in public health facilities in the Gedeo Zone. A randomly selected 577 participants were involved in the study. Data were gathered using an interview-administered, pre-tested, structured questionnaire. The gathered information was imported into Epi Info 3.5.1 and analyzed with SPSS 23. Descriptive data was presented using tables and graphs. A logistic regression model was fitted. A bivariable and multivariable logistic regression model was computed to identify the presence and strength of association. To run multivariable logistic regression analyses, variables with *P*-values of <0.2 were used. The odds ratio, a 95% confidence interval (CI), and a *P*-value of <0.05 were used to identify variables that were associated with primary postpartum hemorrhage.

**Results:**

The magnitude of primary postpartum hemorrhage was 4.2% (95% CI: 2.4–6.0). Postpartum hemorrhage was significantly associated with current antepartum hemorrhage (AOR = 11.67; 95%CI: 7.17–16.17), twin delivery (AOR = 6.59, 95%CI: 1.48–11.70), uterine atony (AOR = 8.45, 95%CI: 4.35–12.55), and prolonged labor (AOR = 5.6, 95%CI: 2.9–8.50).

**Conclusions:**

The prevalence of primary postpartum hemorrhages in the Gedeo Zone, Southern Ethiopia was 4.2%. Current ante partum hemorrhage, twin delivery, uterine atony, and prolonged labor were predictors of primary postpartum hemorrhage. The results back up the necessity for care in the early postpartum period so that clinicians may quickly identify any issues, prevent and start treating excessive blood loss early, and, taking into account the aforementioned factors, possibly reduce the frequency of primary postpartum hemorrhage.

## Introduction

According to global United Nation estimates (2018), 303,000 women worldwide die each year during childbirth or as a result of complications related to pregnancy. Approximately 830 women every day, or one every 2 min, die as a result of this. The largest causes of death are severe bleeding and infections after childbirth, but high blood pressure, obstructed labor, and unsafe abortions also play a role (1). According to statistics from the World Health Organization, 60% of maternal deaths in developing countries are thought to be caused by PPH, accounting for more than 100,000 maternal deaths annually. Several developing nations in the world have maternal mortality rates of more than 1,000 women for every 100,000 live births (2).

Postpartum hemorrhage (PPH) is defined by the World Health Organization (WHO) as blood loss that exceeds 500 mL following a vaginal delivery or 1,000 mL following a cesarean section. Additionally, it is defined as any level of postpartum vaginal bleeding that causes a 10% drop in hemoglobin from the baseline or changed vital signs (3). When PPH occurs within 24 h of delivery, it is referred to as primary PPH; when it happens between 24 h and 6 weeks following delivery, it is referred to as late or secondary PPH (4).

The excessive bleeding that occurs during and after the third stage of labor during PPH puts a woman's life in danger. Up to 18% of deliveries might be affected by it, and it is responsible for 35–55% of peripartum maternal deaths worldwide (5). Over 25% of deaths each year are attributed to it as the main cause of maternal mortality and morbidity worldwide. According to WHO (2015) data, PPH causes more than 100,000 maternal deaths annually worldwide, 60% of which occurs in developing countries (6). Postpartum hemorrhage (PPH) is a common birth complication that typically affects 2–4% of vaginal deliveries and 6% of cesarean deliveries. Uterine atony accounts for more than 50% of PPH cases, followed by retained tissue, genital tract tears, coagulation issues, and uterine rupture (7).

In sub-Saharan Africa, PPH is even higher as a cause of maternal mortality and morbidity. In contrast to developed countries, where pulmonary embolism is the main cause of maternal death, PPH accounts for 25–43% of maternal deaths in developing nations (7). In Ethiopia, evidence revealed that PPH accounts for 54 and 46.5% of maternal deaths in Jimma (8) and Kersa District, Eastern Ethiopia (9), respectively.

The prevalence of PPH, both primary and secondary, was different across developed and developing countries and regions (6, 10–19). For example, it ranges from 6.1% in 2003 to 8.3% in 2011 in New South Wales, Australia (6), and 10.5% in sub-Saharan Africa (19). In addition, it was 9% in Uganda in 2016 (18). A systematic review result in Ethiopia showed that the pooled magnitude of PPH was 8.24% (17). The magnitude of PPH varied from region to region; which was 1.4% (16) in Addis Ababa, 16.6% in the South Nation Nationality People region (11), and 9.4% in the Sidama region (15). A primary PPH occurred in approximately 21.8% of women who gave birth between 2009 and 2013, and a severe primary PPH occurred in 1.4% of cases in Australia (13), 4.28% in Nigeria (14), 2.5% in Afghanistan (12), 16.6% in Southern Ethiopia (11) and 8.8% in North West Ethiopia (10).

Most maternal deaths could have been avoided if women had received proper medical care during their pregnancies and labor, and the key is early diagnosis, which includes risk factors and proper treatment (3). The prolonged third stage of labor, abnormal placentation, use of oxytocin, ante-partum hemorrhage, and hypertension are common risk factors for developing PPH (6, 10–20). Many of these risk factors are detectable during prenatal care or in the first stages of labor, making it appropriate to refer women to a hospital-based institution for prevention and treatment. This is especially important in areas with few resources, which lack suitable preventive options for use during labor and delivery, as well as referral mechanisms and access to emergency obstetric care (21).

Even though maternal death rates occasionally go down, they are remaining high in Ethiopia. The issue is exacerbated in rural areas because there are inadequate facilities and untrained medical personnel to deliver uterotonic injections, the standard treatment for PPH (22). Because of inadequate funding and subpar managerial abilities, public health facilities in developing nations frequently lack the supplies and equipment needed to provide obstetric treatment. Mid-level staff cannot provide essential emergency obstetric treatment due to current ministry policies. PPH continues to be a significant obstacle to maternal health since it is difficult to avoid and treat in low-resource settings due to a lack of competent birth attendants, insufficient blood supply, and a lack of ambulance (23, 24).

The likelihood of dying from PPH is influenced by the woman's overall health as well as the volume, rate, and time of blood loss. The first 24 h are when women are most likely to die from hemorrhage, according to the evidence. Once bleeding begins, death can happen in just 2 h, as opposed to 10 h for eclampsia and 72 h for obstructed labor (25). Early prevention and control of primary PPH's negative effects may be made possible by assessing the prevalence of the condition and identifying its risk factors. The prevention and control program might benefit from risk factor identification. It might also be of utmost relevance to the target audience and lower the expense of its effects. Strategies that are based on data from various healthcare settings and circumstances are required to address these issues. Despite this, there is a paucity of research on the scope of primary PPH and the causes of it throughout Ethiopia, including the study area. Therefore, the findings of this study would provide baseline information about primary PPH and a possible recommendation to policymakers and other stakeholders to design appropriate strategies to decrease the problem. Therefore, this study is aimed at assessing the prevalence of primary postpartum hemorrhage and its associated factors among delivering mothers in Gedeo Zone, Southern Ethiopia, in 2021.

## Materials and methods

### Study setting, design and period

A facility-based cross-sectional study design was deployed in the Gedio Zone from January 1 to March 30, 2021. The Gedeo Zone is one of the administrative zones in the Southern Nations, Nationalities, and Peoples' Region (SNNPR), Ethiopia. The Gedio Zone is located about 369 km from Addis Ababa (capital city of Ethiopia), 90 km to the south of Hawassa (the regional capital), with a total population of 1,105,813 and an area of 1,210.89 square km (26). The Gedeo Zone has twelve woreda, which include Dilla Town, Kochore, Chelelektu Town, Gedeb Town, Chorso, Yirga Chefe Town, Yirga Chefe woreda, Dilla Zuria, Gedeb woreda, Wonago, Bule, and Rappe. It has one referral hospital, three primary hospitals, 38 health centers, 146 health posts, 9 NGO clinics, and private health facilities including low-level clinics, medium clinics, drug stores, and pharmacies that provide health services for the community. Data from the Gedio zone health department has estimated the health service coverage to be 92%. According to the Gedeo Zone health office, there are an estimated 39,424 pregnant women in Gedeo Zone (27).

### Population

All delivering women found in Gedeo Zone public health facilities were the source population, while those women who were present at the selected facility during the data collection period were the study population.

### Inclusion and exclusion criteria

All women who gave birth in all public health facilities during a period of study were included in the study, whereas delivering women who were referred to other health facility were excluded from the study.

### Study variables

Primary postpartum hemorrhage was the dependent variable of the study. The independent variable includes: socio-demographic factors (maternal age, marital status, religion, educational status and occupation); obstetrics factors (parity, ANC utilization, gestational age, twins' pregnancy, prolonged labor, previous history of PPH, uterine atony, retained placenta, had current pregnancy APH, genital tract trauma, obstructed labor); and maternal hemodynamic instability related factors (pulse rate, temperature, blood pressure, respiratory rate and cold extremity).

### Operational definitions

**Primary postpartum hemorrhage:** Postpartum blood loss was visually estimated by the midwives and nurse, during which they made a quantitative estimate of the amount of blood lost. In direct blood collection, all blood lost during the postpartum period (except for the placenta and membranes) is contained in a disposable plastic collector bag, which is attached to a plastic sheet and placed under the woman's buttocks. When the bleeding stops, the bag could be gravimetrically weighed, allowing for a direct measurement (28, 29).

**Hemodynamic instability:** It defined as any instability in the blood which changes (the pulse rate, the respiratory rate, the temperature, the blood pressure, the status of the skin and mucous membranes), which can lead to inadequate arterial blood flow to organs (3).

**Prolonged labor:** It is a failure of labor to progress and can be determined by the labor stage and whether the cervix has thinned and opened appropriately during labor (30).

**Onset of labor:** a series of continuous, progressive contractions of the uterus, additionally characterized by a bloody show and rupture of the amniotic sac (a bag of water), which is self-reported by the parturient or by a clinician report (30).

**Prolonged latent phase of first stage:** It had been defined as a nullipara who has not entered the active phase 20 h after the onset of the latent phase and a multipara who has not entered the active phase 14 h after the onset of the latent phase (30).

**Prolonged in active first stage labor:** A dilatation of cervix <1–2 cm/h after a women reaches the active phase (≥6 cm) is considered a delay in progress of labor (30).

**Prolonged second stage of labor:** this stage covers more than 2.5 h duration for nulliparous and 1 h in multiparous (30).

**Obstructed labor** is defined as labor with little or no progress despite strong uterine contractions confirmed through vaginal and abdominal examination (30).

**Retained placenta:** A placenta that was actively controlled during the third stage of labor and has not undergone placental expulsion within 30 min of the baby's birth (31).

**Uterine atony** is defined as a soft and weak uterus after delivery, and it happens when the uterine muscles don't contract enough to clamp the placental blood vessels shut after childbirth (30, 31).

### Sample size determination and sampling procedure

The sample size was calculated using a formula for estimating the proportion of a single population under the assumption of a 95% confidence level, a 3% margin of error, and a 16.6% prevalence of primary postpartum hemorrhage from the same study conducted in Southern Ethiopia (11). Substituting the above values in the sample size formula, the final sample size of the study was 641, including the 10% non-response rate.

Initially, four districts and one town were selected randomly from all the Gedeo Zone districts, and then all 21 health centers and two primary district hospitals in the selected districts were included in the study. To determine a total of 641 delivered mothers for an exit interview from each of the predetermined health facilities, we used the previous three-month report on the total number of mothers who delivered at the facility, which was 1,475. In addition, a proportional allocation of participants was made between health facilities based on the previous three-month report. Then, a systematic random sampling technique was used with a K-value of 2, and the first study participant was selected randomly.

### Data collection tools and procedures

A structured, pre-tested, and interview-administered questionnaire was used to collect the data. The data collection tool was updated and contextualized to meet the local condition and the research purpose after being adopted from various works of literature (6, 10–19). The questionnaire was prepared in an English version and then translated into local languages (Gedeuffa and Amharic) and finally retranslated into an English version to ensure consistency. Seven interviewers who were health professionals (BSc degree midwives and nurses) and had experience in data collection were hired to collect the data. In order to coordinate the activities of the interviewers, ensure the prompt supply of the necessary materials for interviewers, and check the questionnaire in the field each day, two supervisors (midwives) who were familiar with the population and social administration setting of the districts were hired. The principal investigator was responsible for facilitating the whole process of data collection.

### Data quality assurance

To assure the quality of the data, 2 days of training were given for data collectors and supervisors on the objective of the study, the study tool, and data collection procedures and techniques before the data collection time. Pre-testing of the study tool was carried out among 32 delivering women (5% of the sample size) in Dilla town, which is a similar setting but outside the proposed study area. During pre-testing, the performance of the data collectors, clarity, completeness, and duration of time required to complete each questionnaire were assessed. The collected data was also checked manually for completeness and consistency by supervisors during fieldwork and again before entry. During data entry, a double entry was made to minimize an error.

### Data processing and analysis

Following data collection, the response was coded and entered using Epi Data version 4.2 software before being exported to the Statistical Package for Social Science (SPSS) version 23 for data analysis. Descriptive statistics were presented using percentages, mean, and frequency distribution in tables and figures. Binary logistic regression was fitted to determine the association of independent variables with primary postpartum hemorrhage after the assumption was fulfilled. Variables that had a *p*-value of 0.2 were entered into multivariable analysis. In the final model, adjusted odds ratios (AOR) with 95% CI and *p*-value <0.05 were used to declare the significant variables.

## Results

### Socio-demographic characteristics of respondents

A total of 577 delivering mothers participated in the study, with a response rate of 90.05%. The mean age of mothers was 27 years (SD ± 5.4). The majority (94.8%) of women were married. More than half (61%) of mothers were Protestant believers. In addition, only 27.8% of the respondents had secondary education or above, and 55.5% of women were housewives (Table 1).

**Table 1:** Distribution of delivering women by their socio-demographic characteristics in the district areas health centers of Gedeo Zone, Southern Ethiopia 2021 (*N* = 577).

**Variables**	**categories**	**Frequency**	**Percent**
Age of mother	18–34	460	79.7
	≥35	117	20.3
Marital status	Married	547	94.8
	Not married^*^	30	5.2
Religion	Protestant	352	61
	Orthodox	134	23.2
	Muslim	63	10.9
	Others	28	4.9
Educational status	≤ Primary education^*^	417	72.2
	≥Secondary education	160	27.8
Occupation	House wife	320	55.5
	Merchant	85	14.7
	Daily labor	55	9.5
	Farmer	55	9.5
	Governmental employ	44	7.6
	Others	18	3.1

* Not currently married: Single, divorced and widowed; ≤ Primary education: Unable to read and write, able to read and write and primary education.

### Past obstetric history and current obstetric conditions of respondents

Out of 577 pregnant women, around 3/4th (79.7%) of mothers had at least one ANC follow-up visit during their current pregnancy. Out of them, the majority (78.0%) of the study participants were multiparous (≥2 children), 0.3% had previous cesarean delivery, 2.1% had previous PPH and 2.95% had pre-existing anemia (Table 2).

**Table 2:** Distribution of delivering women by their past obstetric history in the district areas health centers of Gedeo Zone, Southern Ethiopia, 2021 (*N* = 577).

**Variables**		**Frequency**	**Percent**
ANC visit during current pregnancy	Yes	460	79.7
	No	117	20.3
Parity	I	127	22.0
	II-IV	300	52.0
	≥V	150	26.0
History of abortion	Yes	49	8.5
	No	528	91.5
History of still birth	Yes	14	2.4
	No	563	97.6
History of anemia	Yes	17	2.95
	No	560	97.05
History of Cesarean section	Yes	2	0.3
	No	575	99.6
Previous history of PPH	Yes	20	3.5
	No	557	96.5
Uterine curettage	Yes	28	4.9
	No	549	95.1

ANC, Ante natal care; PPH, Postpartum hemorrhage.

Among the total study participants, all of them gave birth through spontaneous vaginal delivery, and episiotomy was done for 30.8% of respondents. About 17.9% of women had prolonged labor and 7.5% had obstructed labor. During ANC follow-up, antepartum hemorrhage was detected in 5.9% of mothers and in twin pregnancy (1.7%). Among fifty-six women who were facing genital tract trauma, 5.2% had vaginal wall laceration and 3.8% had perennial tears (Table 3).

**Table 3:** Distribution of delivering women by their current obstetric condition in the district areas health centers of Gedeo Zone, Southern Ethiopia, 2021 (*N* = 577).

**Variable response**		**Frequency**	**Percent**
Prolonged labor	Yes	103	17.9
	No	474	82.1
Obstructed labor	Yes	43	7.5
	No	534	92.5
Delivery characteristic	Single	567	98.3
	Twins	10	1.7
History of APH in current pregnancy	Yes	34	5.9
	No	543	94.1
Baby weight in gm	<2,500	15	2.6
	2,500–3,999	381	66.0
	>4,000	53	9.2
	Unknown	128	22.2
Instrumental delivery	Yes	27	4.7
	No	550	95.3
Induction/augmentation of labor	Yes	167	28.94
	No	410	71.06

### Maternal hemodynamic conditions

Out of a total of 24 mothers who developed PPH, 16 (48.5%) of the respondents had hypotension (orthostatic type), 17 (63.0%) tachypnoea, 14 (63.6%) tachycardic, 16 (51.6%) hypothermic, and 16 (40%) of them had cold extremities but there was no maternal death reported in this study (Table 4).

**Table 4:** Distribution of delivering women by their hemodynamic condition in the district areas health centers of Gedeo Zone, Southern Ethiopia, 2021 (*N* = 577).

**Hemodynamic measurement**		**Yes**		**No**	
		**Number**	**%**	**Number**	**%**
Blood pressure	<90/60	16	48.5%	17	17.9%
	90/60–120/80	6	1.3%	467	98.7%
	>120/80	2	2.8%	69	97.3%
Pulse rate	<60	5	26.3%	14	19%
	60–100	5	0.9%	531	536$
	>100	14	63.6%	8	22%
Respiratory rate	<18	1	5.6%	17	94.4%
	18–24	6	1.1%	526	98.9%
	>24	17	63.0%	10	37.0%
Temperature	<35.5°c	16	51.6%	15	48.4%
	35.5–37.7°c	5	1.0%	511	99.0%
	>37.7°c	3	10%	27	90.0%
Cold extremity	Yes	16	40.0%	24	60.0%
	No	8	1.5%	529	98.5%

### Magnitudes of postpartum hemorrhage

**Figure 1:**
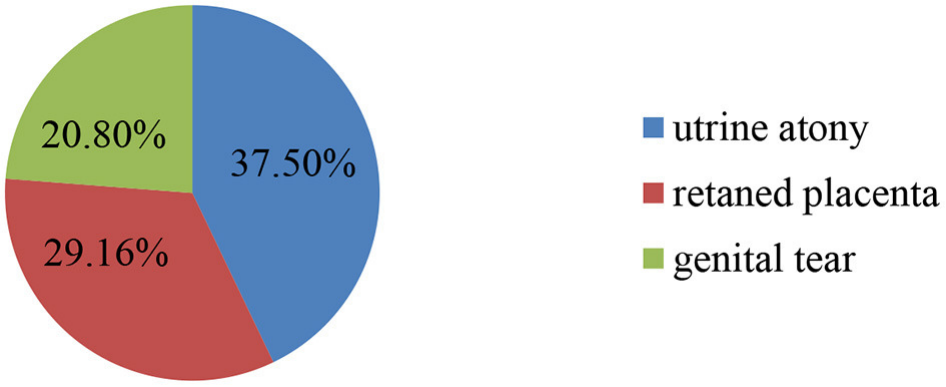
Causes of PPH in women who give birth in the district areas health centers of Gedeo Zone, Southern Ethiopia, 2021 (*N* = 577).

About 24 cases of PPH were found during the study period, which put the magnitude of primary PPH at 4.2% (95% CI: 2.4–6.0). The causes of PPH identified during the study period were uterine atony (37.5%), followed by the retained placenta (29.16%), and genital tear (20.8%) (Figure 1).

### Factors associated with PPH

In the bivariable analysis, variables having a *p*-value of <0.2 such as maternal age, educational status, parity, ANC follow up, multiple pregnancies, current APH, history of previous PPH, prolonged labor, obstructed labor, retained placenta, and uterine atony were candidates for multi variable analysis. The Hosmer and Lemeshow test result (0.84) indicated the model displays a good fit for analysis. However, the result of the multivariable analysis showed only four variables, namely current APH, uterine atony, delivery characteristics, and prolonged labor, were significantly associated with primary PPH.

Accordingly, multivariate analysis has shown that delivering women who had current APH were around twelve times more likely to develop primary PPH than those mothers who had no antepartum hemorrhage (AOR = 11.67; 95% CI: 7.17–16.17). Delivering women who gave birth to twin babies were almost seven times more likely to develop primary PPH as compared to those who gave birth to a single baby (AOR = 6.59, 95% CI: 1.48–11.70). In addition, those who have uterine atony were eight times more likely to develop primary PPH when compared to their counterpart (AOR = 8.45, 95% CI: 4.35–12.55). Furthermore, delivering women who had a prolonged 3rd stage of labor were five times more likely to develop primary PPH as compared to those who had a normal 3rd stage of labor (AOR = 5.6, 95% CI: 2.9–8.50) (Table 5).

**Table 5:** Factors associated with PPH among delivering women who give delivery in the district areas health centers of Gedeo Zone, Southern Ethiopia, 2021 (*N* = 577).

**Variables**	**Primary PPH**		**COR (95%CI)**	**AOR (95%CI)**
	**Yes**	**No**		
**Current APH**				
Yes	11	23	19.49 (7.88–48.14)	11.67 (7.17–16.17)^***^
No^*^	13	530	1	1
**Delivery characters**				
Single^*^	21	546	1	1
Twine	3	7	11.14 (7.44–14.84)	6.59 (1.48–11.70)^**^
**Uterine atony**				
Yes^*^	9	15	21.52 (12.52–30.52)	8.45 (4.35–12.55)^***^
No	15	538	1	1
**Prolonged labor**				
Yes	19	84	21.22 (17.74–24.70)	5.60 (2.9–8.50)^***^
No^*^	5	469	1	1

APH, Antepartum hemorrhage; COR, Crud odds ratio; AOR, Adjusted odds ratio.

*** *P* < 0.01;

** *P* < 0.05.

## Discussion

In the current study, the overall prevalence of primary PPH was 4.2%. It was relatively lower compared to studies done in Australia (21.8%) (13), Pakistan (21.3%) (32), India (9.2%) (33), Tanzania (11.9%) (34), Cameron (23%) (35), Uganda (9%) (18), Nigeria (4.28%) (14) and Ethiopia (5.8–18%) (10, 11, 36, 37). This variation in our study may be caused by differences in study design, social stability, intercultural compatibility, and accessibility of maternal healthcare services. Additionally, the prevalence of PPH may change over the course of the study and within and between geographic locations. An additional reason might be the difference in policies and strategies designed by the countries or regions toward maternal and child health. Furthermore, the sample size may also be the reason for the difference.

However, when compared to a study conducted in India with a prevalence of 1.07% (7) and 3.4% (38), Afghanistan (2.5%) (12), and some low-resource setting countries (1.6%) (39), the results of this study were the highest. The reasons for these discrepancies might be the variation in study settings, study designs, and study periods. The similarities and differences in magnitude of primary PPH in the above studies might be due to the criteria used to define primary PPH. Additionally, this discrepancy can be a sign of the inefficiency of national plans for providing services for maternal health.

The results of this study showed that the likelihood of primary postpartum hemorrhage was high among women with APH, uterine atony, twin delivery, and prolonged labor. Almost all of these described factors agree with the findings of many other studies conducted both in developed and developing countries (2, 6, 10–19, 32, 35, 36, 39, 40). Therefore, these similarities implied that those factors might be the common contributors to the occurrence of primary postpartum hemorrhage.

Accordingly, in this study, the odds of primary PPH were higher among women with uterine atony. The finding was comparable with a study conducted in Norway (60%) (41), Afghanistan (65.6%) (12), Nigeria (14), Cameroon (35), Uganda (18), and low resource setting countries (39), including Southern Ethiopia (15). The reason may be a difference in practice of management of the 3rd stage of labor and a difference in the skills of the birth attendant. The reasons for the similarity might be that most of the studies are found in the Sub-Saharan African regions with similar populations and living standards, and the studies were conducted in similar study settings. Another justification could be that Ethiopia occasionally uses more institutional deliveries, which reduces the risk of PPH by removing any retained products and supplying the uterus with oxytocin to trigger contractions.

In the current study, prolonged labor was one of the significant factors for primary PPH. In a similar manner, this finding is supported by research done at Bonassama Hospital in Cameroon (39) and Afghanistan (12). This result was consistent with earlier findings from Ethiopia (10, 15). This might be explained by the possibility that the pronged labor raises the danger of laceration to the pelvic blood vessels and soft tissue. This significantly lessens uterine contraction. As a result, there may be a significant increase in the likelihood of blood loss following delivery.

Furthermore, this study discovered that antepartum hemmorrage in the current pregnancy was one of the major contributors to primary postpartum hemmorhage. Similarly, it was consistent with a study conducted in Ethiopia (11) and Afghanistan (12). The findings of those studies showed that cervical or vaginal laceration and antepartum hemorrhage in a recent pregnancy were risk factors for the primary PPH.

Furthermore, the women's twin births had accelerated the development of PPH. The finding is supported by other research (32, 36). This may be because stretching of muscle fibers during twin pregnancy causes loss of muscle tone, which causes uterine atony and puts the mother at risk for various issues.

### Strength and limitation

This study was the first in the area and included a wide area to make the study representative, which could be considered a strength. Studies like this one may have limitations related to blood loss measurement because it is subjective and often erroneous during labor, especially for greater quantities that are more likely to be underestimated. The study would not be representative of the catchment area since women who gave birth at home were not surveyed due to socio-cultural issues and associated misconceptions about maternal health services. The study evaluates the past obstetric histories of the mothers, which could lead to recall biases. The inability to demonstrate a cause-and-effect link is another drawback of this study's design, and variances may also be seen because different clinicians with varying levels of training, experience, and grade made the diagnosis of primary PPH.

## Conclusions and recommendations

The prevalence of primary postpartum hemorrhage in the study area was 4.2%. Antepartum hemorrhage, multiple pregnancies, uterine atony, and prolonged labor were all associated with postpartum hemorrhage in a significant and independent way. Our research suggests that early postpartum care is necessary to enable clinicians to quickly identify and start treating excessive blood loss. Healthcare personnel should monitor the labor's development and take all required actions when they are needed. Additionally, early identification of possible risks for pregnancy-related problems and adherence to all recommended interventional guidelines are essential steps to reduce the likelihood of developing PPH. Therefore, the occurrence of postpartum hemorrhage in the Gedeo Zone, Southern Ethiopia needs to be a significant health concern, and postpartum care of women is essential, especially for those with the above factors that require more attention. It is also advised to do research while taking into account elements of the health system, service providers, and cultural context.

## Data availability statement

The original contributions presented in the study are included in the article/supplementary material, further inquiries can be directed to the corresponding author.

## Ethics statement

The study involving human participants were reviewed, approved, and Ethical approval was obtained from the Dilla University College of Medicine and Health Science institutional review board (IRB).

## Author contributions

GZ and WZ: conceptualization, data gathering, formal analysis, methodology, software, supervision, first draft writing, and writing review and editing. GZ, TE, SD, and WZ: formal analysis, methodology, validation, visualization, and first draft writing. All authors contributed to the article and approved the submitted version.
